# Medical Service Use and Charges for Cancer Care in 2018 for Privately Insured Patients Younger Than 65 Years in the US

**DOI:** 10.1001/jamanetworkopen.2021.27784

**Published:** 2021-10-06

**Authors:** Nicholas G. Zaorsky, Chachrit Khunsriraksakul, Samantha L. Acri, Dajiang J. Liu, Djibril M. Ba, John L. Lin, Guodong Liu, Joel E. Segel, Joseph J. Drabick, Heath B. Mackley, Douglas L. Leslie

**Affiliations:** 1 Department of Radiation Oncology, Penn State Cancer Institute, Hershey, Pennsylvania; 2 Department of Public Health Sciences, Penn State College of Medicine, Hershey, Pennsylvania; 3 Department of Health Policy and Administration, Pennsylvania State University, University Park; 4 Penn State Cancer Institute, Hershey, Pennsylvania; 5 Department of Medical Oncology, Penn State Cancer Institute, Hershey, Pennsylvania; 6 Department of Radiation Oncology, Geisinger Health System, Danville, Pennsylvania

## Abstract

**Importance:**

Currently, there are limited published data regarding resource use and spending on cancer care in the US.

**Objective:**

To characterize the most frequent medical services provided and the associated spending for privately insured patients with cancer in the US.

**Design, Setting, and Participants:**

This cohort study used data from the MarketScan database for the calendar year 2018 from a sample of 27.1 million privately insured individuals, including patients with a diagnosis of the 15 most prevalent cancers, predominantly from large insurers and self-insured employers. Overall societal health care spending was estimated for each cancer type by multiplying the mean total spending per patient (estimated from MarketScan) by the number of privately insured patients living with that cancer in 2018, as reported by the National Cancer Institute’s Surveillance, Epidemiology, and End Results program. Analyses were performed from February 1, 2018, to July 8, 2021.

**Exposures:**

Evaluation and management as prescribed by treating care team.

**Main Outcomes and Measures:**

*Current Procedural Terminology* and Healthcare Common Procedure Coding System codes based on cancer diagnosis code.

**Results:**

The estimated cost of cancer care in 2018 for 402 115 patients with the 15 most prevalent cancer types was approximately $156.2 billion for privately insured adults younger than 65 years in the US. There were a total of 38.4 million documented procedure codes for 15 cancers in the MarketScan database, totaling $10.8 billion. Patients with breast cancer contributed the greatest total number of services (10.9 million [28.4%]), followed by those with colorectal cancer (3.9 million [10.2%]) and prostate cancer (3.6 million [9.4%]). Pathology and laboratory tests contributed the highest number of services performed (11.7 million [30.5%]), followed by medical services (6.3 million [16.4%]) and medical supplies and nonphysician services (6.1 million [15.9%]). The costliest cancers were those of the breast ($3.4 billion [31.5%]), followed by lung ($1.1 billion [10.2%]) and colorectum ($1.1 billion [10.2%]). Medical supplies and nonphysician services contributed the highest total spent ($4.0 billion [37.0%]), followed by radiology ($2.1 billion [19.4%]) and surgery ($1.8 billion [16.7%]).

**Conclusions and Relevance:**

This analysis suggests that patients with breast, colorectal, and prostate cancers had the greatest number of services performed, particularly for pathology and laboratory tests, whereas patients with breast, lung, lymphoma, and colorectal cancer incurred the greatest costs, particularly for medical supplies and nonphysician services. The cost of cancer care in 2018 for the 15 most prevalent cancer types was estimated to be approximately $156.2 billion for privately insured adults younger than 65 years in the US.

## Key Points

**Question:**

What are the most common and costly medical procedures and services provided to privately insured patients with cancer in the US?

**Findings:**

This cohort study found that the total estimated cost of cancer care for privately insured adults in the US was $156.2 billion. Patients with breast, colorectal, and prostate cancers had the greatest number of services performed, particularly for pathology and laboratory tests, and patients with breast, lung, and colorectal cancer incurred the highest costs, particularly for medical supplies and nonphysician services.

**Meaning:**

This study suggests that, for privately insured patients with cancer, pathology and laboratory services contributed the most to the number of services performed, but medical supplies and nonphysician services contributed the most to spending.

## Teaser

This cohort study uses 2018 data from the MarketScan database to characterize the most frequent medical services provided and the associated spending for privately insured patients with cancer in the US.

## Introduction

Cancer care spending in the US for those older than 65 years with Medicare was estimated to be $125 billion in 2010; this amount is estimated to increase to $158 billion in 2020.^1,2^ The largest increases are seen in the continuing phase of care for prostate cancer (42%) and female breast cancer (32%). National spending for cancer care is substantial and expected to increase because of population changes alone.

The specific expenses that contribute to these figures are poorly understood. The direct costs of cancer care include diagnostic tests, hospital and physician fees, and the cost of drug therapy. Meropol and Schulman^3^ describe how the high price of new drugs obscures other direct costs that are more difficult to enumerate. Furthermore, in the European Union, cancers of the lung, breast, prostate, and colorectum have been shown to contribute to the plurality of cost, with most costs stemming from inpatient care.^4^ In the US, many patients have private insurance or are diagnosed before 65 years of age. To our knowledge, there are limited data regarding population estimates of resources and spending on cancer care in the US for patients with private insurance.

The purpose of the current work is to characterize the most frequent medical services provided for privately insured cancer patients in the US and the costs associated with these services. The results of this work may be used to identify patients and services that most contribute to spending, with the ultimate goal of identifying potential targets for decreasing resource consumption.

## Methods

The IBM Watson Health MarketScan database was used to summarize expenditures reimbursed to the clinician for care. MarketScan is a private insurance claims database. This retrospective cohort study used data from the MarketScan database for the calendar year 2018 from a sample of 27.1 million privately insured individuals, including 402 115 patients with a diagnosis of the 15 most prevalent cancers, predominantly from large insurers and self-insured employers. Expenditures reimbursed to the clinician for care include the amount paid by insurance companies and patient out-of-pocket payments, including copays and deductibles. Payments were taken from the most recently available year, 2018, for the 15 cancers with the highest incidence as defined by *International Statistical Classification of Diseases and Related Health Problems, Tenth Revision* codes. We limited our analysis to adult patients younger than 65 years at the time of cancer diagnosis in 2018. Based on previous work,^5,6^ it is estimated that the median age of patients with a new diagnosis of cancer is 65 years (interquartile range [IQR], 55-74 years). This research project was approved by the Penn State College of Medicine institutional review board, which waived the requirement for informed consent because deidentified data were used. This study followed the Strengthening the Reporting of Observational Studies in Epidemiology (STROBE) reporting guideline.

For each of the top 15 cancers, the *Current Procedural Terminology* (*CPT*) codes were subdivided into the following categories: anesthesia (eg, anesthesia procedure for endoscopy), surgery (eg, mastectomy and prostatectomy), radiology (eg, chest radiograph and computed tomography), pathology and laboratory (eg, biopsy and basic metabolic profile), medical services (eg, chemotherapy administration), evaluation and management services (eg, outpatient or inpatient visits), and medical supplies and nonphysician services (eg, dexamethasone and ondansetron injections). The medical supplies and nonphysician services category was retrieved according to the Healthcare Common Procedure Coding System (HCPCS) level II codes. Codes associated with each category are provided in eTable 1 in the Supplement.

### Statistical Analysis

Analyses were performed from February 1, 2018, to July 8, 2021. To extrapolate to the overall population in the US, total health care spending was estimated for each cancer type by multiplying the mean total spending per patient from MarketScan by the number of patients living with that cancer in 2017, as reported by the National Cancer Institute’s Surveillance, Epidemiology, and End Results (SEER) program.^7,8,9^ At the time of the analysis, cancer prevalence in 2017 was the most recent version provided by SEER. Because previous work estimated that 41.8% of patients with cancer have private insurance, the costs for all privately insured patients with cancer in the US could be calculated by multiplying 0.418 by the estimated costs for all cancer patients in the US.^6^ Data were analyzed using R, version 3.6.3 (R Group for Statistical Computing) and Illustrator (Adobe Inc).

## Results

**Figure 1.**
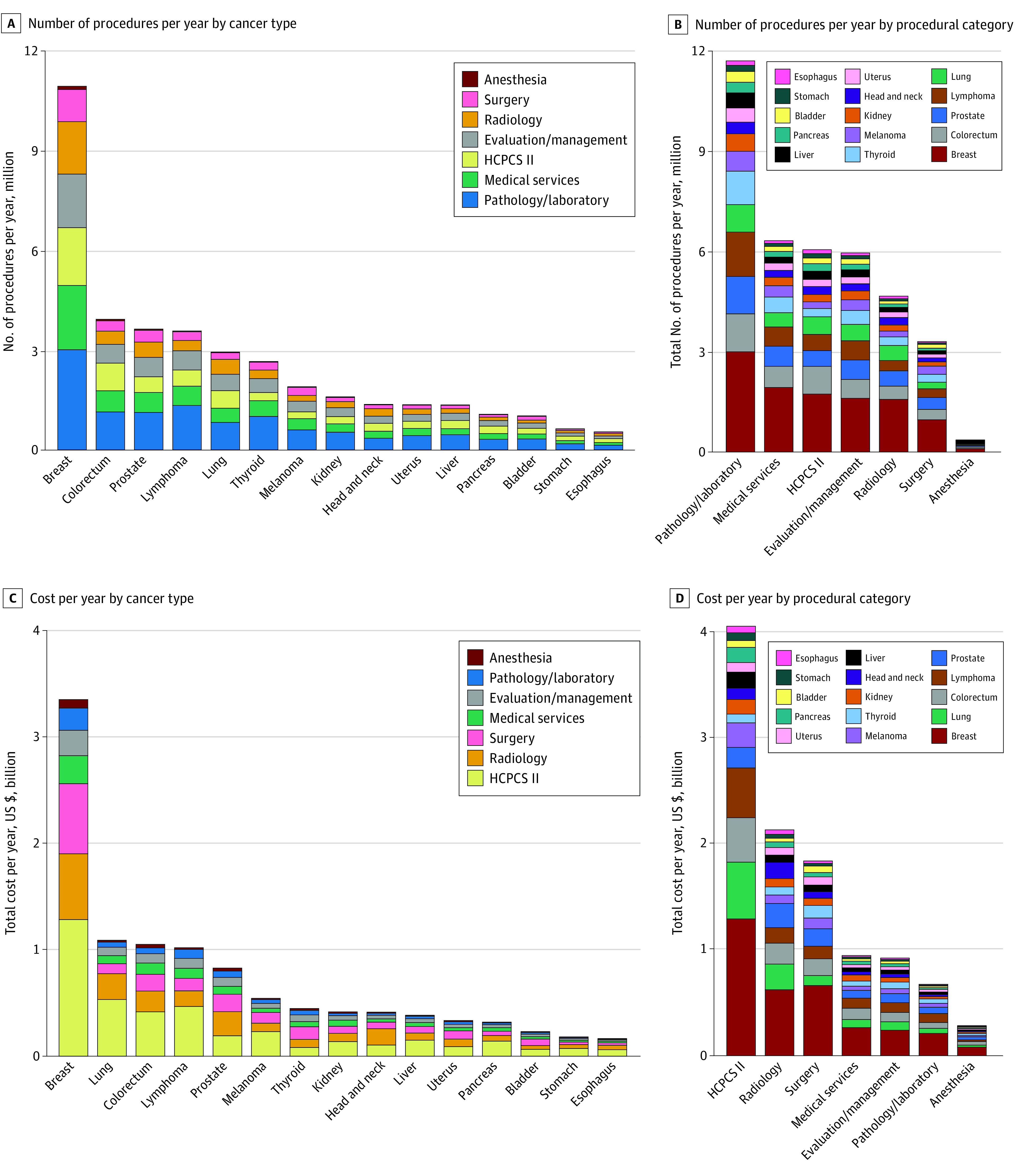
Total Number of Procedures and Amount Spent Based on a Nationally Representative Sample From the MarketScan Database A, Total number of procedures per year plotted against the top 15 cancers identified using *International Statistical Classification of Diseases and Related Health Problems, Tenth Revision* (*ICD-10*) codes. The plurality of procedures within all cancer categories were mainly attributable to pathology and laboratory services. B, Total number of procedures in one calendar year was plotted against the procedural categories identified by *Current Procedural Terminology* (*CPT*) or Healthcare Common Procedure Coding System (HCPCS II) codes. The pathology and laboratory category accounted for most procedures. C, Total spent per year plotted against the top 15 cancers identified using *ICD-10* codes. Most of the cost for breast and lung cancer was from medical supplies and nonphysician services (HCPCS II code). D, Total spent per year plotted against the top procedural categories identified by *CPT* codes. HCPCS II, radiology, and surgery contributed to the majority of costs.

Among 27.1 million patients enrolled in the MarketScan database in 2018, there were 402 115 patients with cancer with 38.4 million documented procedure codes for the 15 most common cancers, with a total cost of $10.8 billion. The top 5 most common cancers according to the MarketScan database included breast cancer (n = 124 543), prostate cancer (n = 56 775), thyroid cancer (n = 40 974), lymphoma (n = 31 760), and colorectal cancer (n = 30 942) (Table). The median number of services was 1.6 million (IQR, 1.2 million-3.3 million) per cancer type, with median spending of $419.2 million (IQR, $330.0 million-$928.2 million) per cancer type. Figure 1 shows a breakdown of the number of services performed for the cancers and their spending. eTable 1 in the Supplement lists the most common *CPT* codes by group along with mean spending per service and median spending. eTable 2 in the Supplement lists the costliest *CPT* codes by group, along with mean spending per service and median spending. eTable 3 in the Supplement provides these data by procedure group.

**Table.** Median Numbers of Procedures and Expenditures and Estimated Total Expenditure per Cancer Type Based on MarketScan and SEER Databases^a^

Cancer	No. of patients in sample from MarketScan	Total spent in MarketScan database, $	Median (IQR) spent per patient, $	No. of procedures per patient, median (IQR)	Prevalence based on SEER data	Estimated total spent in US, $	
						For calendar year 2018	For adults aged <65 y privately insured for calendar year 2018
Breast	124 543	3 365 187 055	6601 (2220-25 464)	28 (17-50)	3 597 331	97 200 900 190	40 629 976 279
Prostate	56 775	832 091 774	4722 (1418-13 640)	24 (14-39)	3 170 339	46 464 341 754	19 422 094 853
Colorectum	30 942	1 055 990 841	10 613 (3704-34 768)	34 (19-61)	1 348 087	46 007 611 823	19 231 181 742
Lung	16 882	1 094 695 214	27 334 (8089-84 563)	53 (29-83)	558 250	36 199 123 527	15 131 233 634
Lymphoma	31 760	1 024 229 269	7803 (2393-29 007)	30 (17-56)	935 362	30 164 519 447	12 608 769 129
Melanoma	28 989	546 929 875	5008 (2186-12 450)	25 (16-39)	1 245 276	23 494 382 233	9 820 651 774
Uterus	14 254	337 649 728	7944 (2312-25 257)	32 (17-56)	793 846	18 804 678 390	7 860 355 567
Head and neck	12 238	416 732 551	9490 (3003-35 785)	31 (17-58)	479 647	16 333 103 295	6 827 237 177
Bladder	11 302	233 378 184	8242 (3090-19 543)	31 (18-51)	712 613	14 714 946 716	6 150 847 727
Kidney	16 087	419 240 007	8612 (3133-21 634)	33 (19-54)	558 023	14 542 522 922	6 078 774 582
Thyroid	40 974	451 186 487	4017 (1525-11 458)	26 (16-42)	859 838	9 468 133 130	3 957 679 648
Stomach	3048	182 774 668	29 821 (9134-79 329)	61 (32-92)	116 525	6 987 473 151	2 920 763 777
Liver	6444	387 129 448	30 156 (9448-80 520)	62 (34-93)	89 949	5 403 771 987	2 258 776 690
Pancreas	5162	322 470 211	32 806 (10 866-85 326)	60 (34-90)	78 969	4 933 194 509	2 062 075 305
Esophagus	2715	167 300 995	29 864 (8677-82 998)	57 (28-90)	47 689	2 938 643 522	1 228 352 992

Abbreviations: IQR, interquartile range; SEER, Surveillance, Epidemiology, and End Results.

^a^ MarketScan data from calendar year 2018 were used. Prevalence data from SEER were based on 2017 data because 2018 data were not yet available at the time of the analysis. Prevalence data between 2017 and 2018 are similar.

Patients with breast cancer contributed the greatest total number of services (10.9 million [28.4%]), followed by those with colorectal cancer (3.9 million [10.2%]) and prostate cancer (3.6 million [9.4%]) (Figure 1A). Among the 7 categories of services, pathology and laboratory tests contributed the highest number of services performed (11.7 million [30.5%]), followed by medical services (6.3 million [16.4%]) and medical supplies and nonphysician services (6.1 million [15.9%]) (Figure 1B). The costliest cancers were those of the breast ($3.4 billion [31.5%]), followed by lung ($1.1 billion [10.2%]) and colorectum ($1.1 billion [10.2%]) (Figure 1C). Among the 7 categories of services, medical supplies and nonphysician services contributed the highest total spent ($4.0 billion [37.0%]), followed by radiology ($2.1 billion [19.4%]) and surgery ($1.8 billion [16.7%]) (Figure 1D).

**Figure 2.**
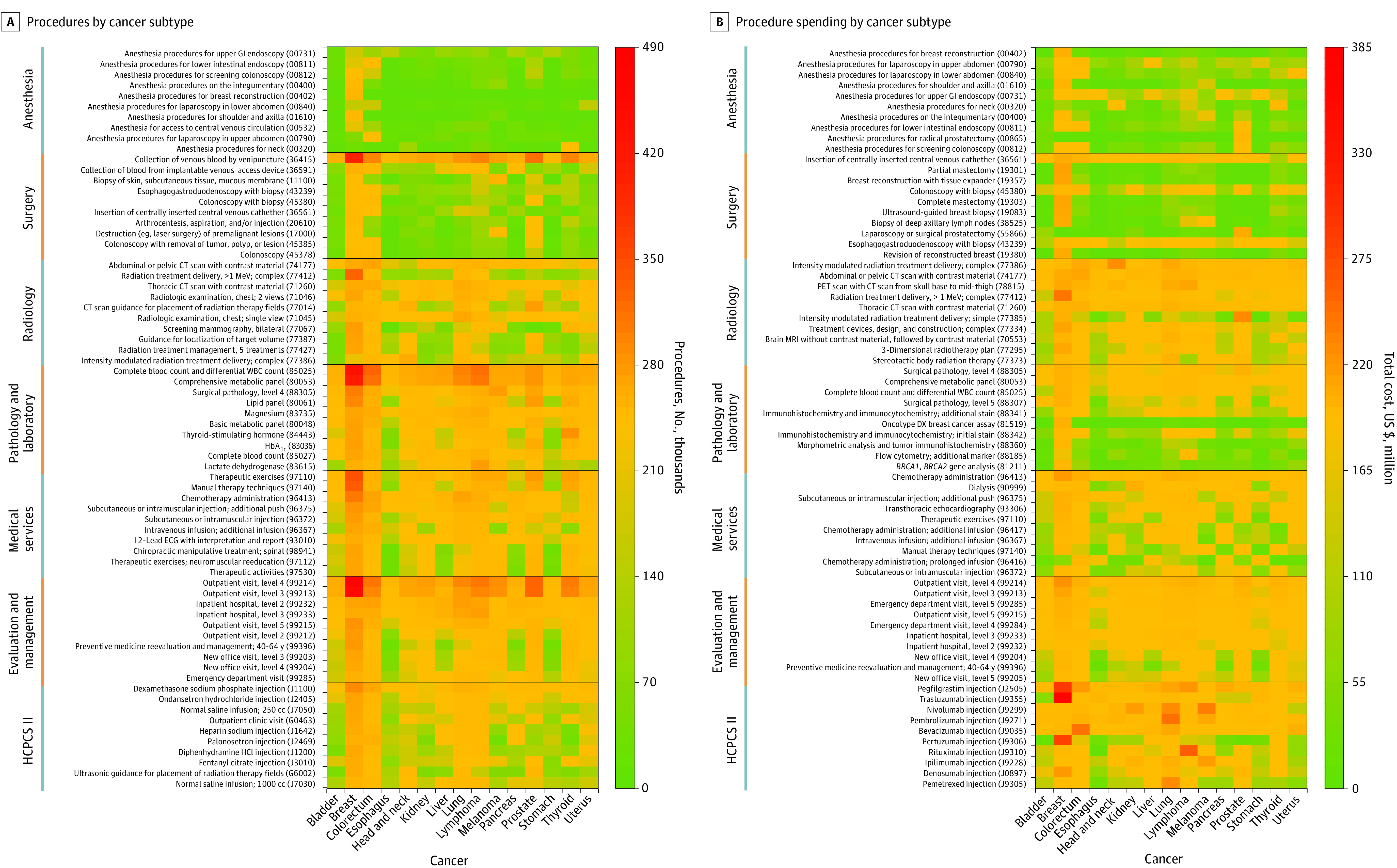
Heat Maps of Individual Procedure Codes and Their Associated Spending A, Total number of codes in one calendar year was plotted against the cancer subtype. Outpatient hospital visits contributed to the greatest number of codes. B, Total spending per year associated with each *Current Procedural Terminology *code plotted against the cancer subtype. CT indicates computed tomography; DX, diagnosis; ECG, electrocardiography; GI, gastrointestinal; HbA_1c_, hemoglobin A_1c_; HCPCS II, Healthcare Common Procedure Coding System; MRI, magnetic resonance imaging; PET, positron emission tomography; and WBC, white blood cell.

Heat maps of the top 10 *CPT* and HCPCS codes from each procedural category among the top 15 cancer subtypes are illustrated in Figure 2. Outpatient hospital visits, level 3 to 4 (ie, office or other outpatient visit, which are 15 or 25 minutes, respectively) were the most commonly billed codes for all cancers (2.8 million performed in 2018), particularly for breast and prostate cancers (Figure 2A). Pegfilgrastim and trastuzumab injections contributed the highest amount spent, particularly for breast cancer (median cost, $22 428 [IQR, $13 276-$36 531] and $53 004 [IQR, $23 655-$90 633], respectively) (Figure 2B).

**Figure 3.**
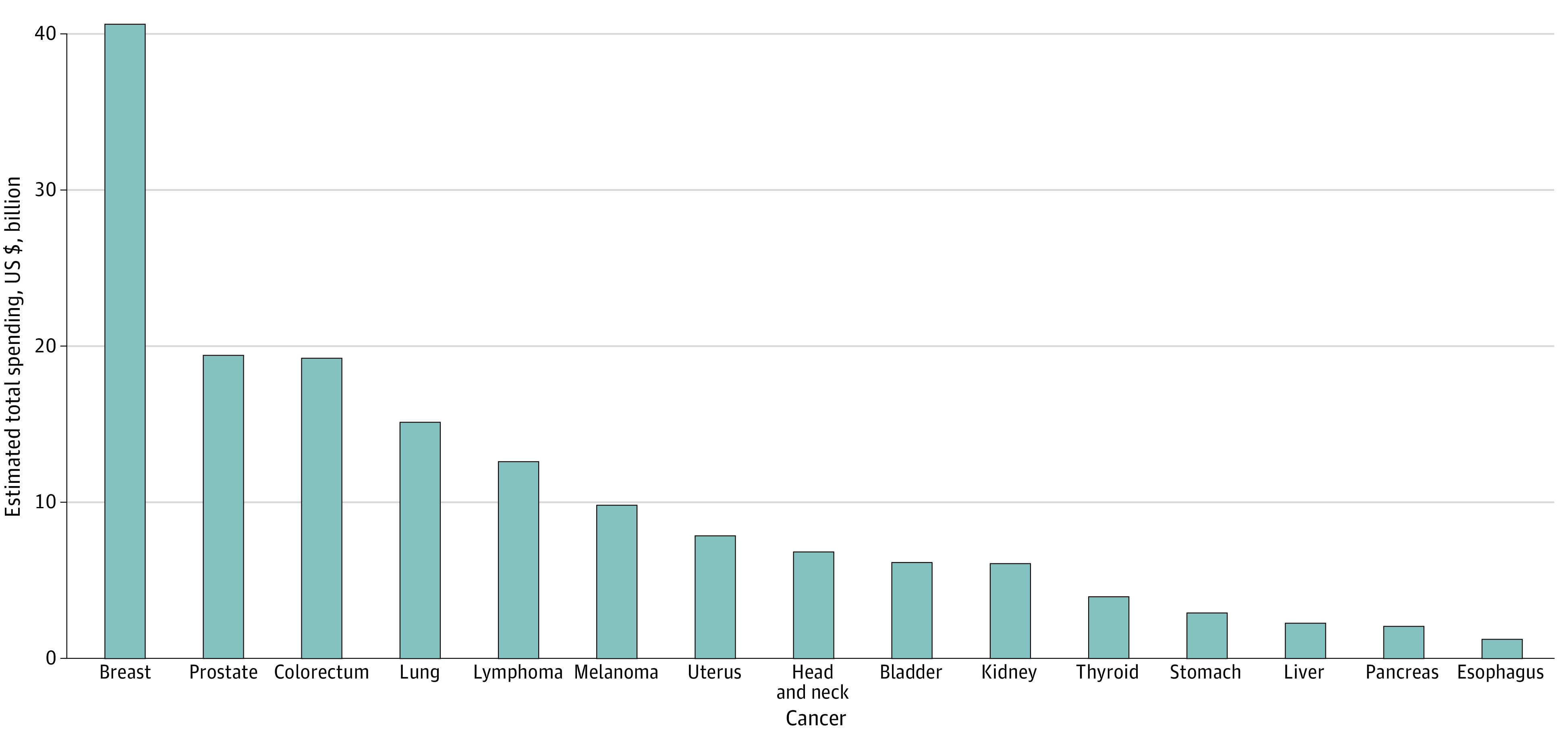
Estimated Total Spending on Cancer Care for Privately Insured Patients With Cancer in the United States in 2018 Total societal spending of care by cancer type for privately insured patients with cancer who were younger than 65 years in the United States.

The Table lists the median number of procedures performed per patient, median spent per patient, and total spending in the MarketScan database and further illustrates the estimated total spending in the US. According to the MarketScan data, the top 3 cancers with the highest median number of procedures performed per patient were liver cancer (median, 62 [IQR, 34-93]), stomach cancer (median, 61 [IQR, 32-92]), and pancreatic cancer (median, 60 [IQR, 34-90]). The median spent per patient was the highest for pancreatic cancer (median, $32 806 [IQR, $10 866-$85 326]), followed by liver cancer (median, $30 156 [IQR, $9448-$80 520]) and esophageal cancer (median, $29 864 [IQR, $8677-$82 998]). In contrast, the estimated total spending for privately insured patients with cancer in the US was the highest for patients with breast cancer ($40.6 billion), followed by prostate cancer ($19.4 billion) and colorectal cancer ($19.2 billion). This inconsistency is owing to the highest prevalence of breast, prostate, and colorectal cancers according to the estimates from the SEER database. MarketScan data showed that patients with breast, colorectal, and prostate cancer had the greatest number of services performed, and the plurality of these services were attributable to pathology and laboratory tests (27.5%, 29.0%, and 30.9% of total services, respectively). Estimates of the overall health care spending for each cancer type for privately insured patients with cancer in the US are further summarized in Figure 3. The cost of cancer care in 2018 for the 15 most prevalent cancer types was estimated to be approximately $156.2 billion for privately insured adults younger than 65 years.

## Discussion

To our knowledge, this is the first characterization of service consumption and spending for privately insured adult patients younger than 65 years with cancer in the US. The overall annual health care spending for privately insured patients with the 15 most prevalent cancers is $156.2 billion. MarketScan data showed that patients with breast, colorectal, and prostate cancer had the greatest number of services performed, and the plurality of these services were attributable to pathology and laboratory tests (27.5%, 29.0%, and 30.9% of total services, respectively). On the other hand, the costliest cancers were those of the breast, lung, and colorectum. Medical supplies and nonphysician services (37.0% of total spending) contributed most to the plurality of spending of patients with cancer, followed by radiology (19.4% of total spending) and surgery (16.7% of total spending). More research is needed to better identify and target wasteful procedures.

In 2011, Mariotto et al^2^ characterized the cost of initial cancer treatment for US Medicare patients (ie, those ≥65 years of age) diagnosed through 2006, and estimated projections from 2010 to 2020. Similar to our work, they found that breast cancer, lung cancer, colorectal cancer, lymphoma, and prostate cancer were among the costliest forms of cancer. These cancers were projected to be costliest in part because of their relatively high incidence,^10,11,12^ use of multimodality care (ie, surgery, systemic therapy, and radiotherapy), and rising cost of cancer-specific drugs within each disease site.^13,14,15^ Oncology drugs account for the largest spending of any specialty,^13^ and the US accounts for about half of the $100 billion spent worldwide on drugs.^15,16^ From 2010 to 2020, the total expenditure for cancer care is estimated to increase by 26%,^2^ with the cost of drugs increasing by more than 50%.^17^ The total cost for 2020 in the analysis by Mariotto et al^2^ was projected to be $207 billion, assuming a 5% annual increase in the mean costs of care.

The analysis by Mariotto et al^2^ is complementary to our work, and the estimated value differs slightly from ours for several reasons. First, in that work, the authors evaluated patients 65 years of age or older with Medicare, whereas, in our work, patients were younger than 65 years and privately insured. Material financial hardship is more common among cancer survivors aged 18 to younger than 65 years than among those aged 65 years or older (28.4% vs 13.8%; *P* < .001).^18^ Although cancer is less common among those younger than 65 years, and overall costs may be lower in this population, younger patients with cancer have a longer expected survival,^12,19^ and have higher costs throughout their longer follow-up period.^20^ Second, 17 cancers were evaluated in the analysis by Mariotto et al^2^ vs 15 cancers in our analysis; however, these 15 cancers represent more than 90% of all cancer diagnoses. Third, Mariotto et al^2^ considered 3 phases of care: the first 12 months after diagnosis, the final 12 months of life, and all the months in between those 2 phases; in contrast, our analysis is a snapshot of spending in 1 calendar year, 2018. Finally, Mariotto et al^2^ made projections from 2010 to 2020 assuming a constant annual increase in the cost of care. This assumption is unlikely owing to the introduction of many novel and expensive treatments over time, such as the approval of pembrolizumab in 2016 for metastatic non–small cell lung cancer.^21^

Tangka et al^22^ performed a comprehensive analysis of how aggregate cancer costs changed over time. In 2001 to 2005, the shares of cancer costs were as follows: private insurance, 50%; Medicare, 34%; out-of-pocket payments, 8%; other public insurance, 5%; and Medicaid, 3%. Cancer-related treatment costs shifted away from the inpatient setting and toward the outpatient setting, and the share of these costs paid for by private insurance and Medicaid has increased—the share of total cancer costs that resulted from inpatient admissions decreased from 64.4% in 1987 to 27.5% in 2001 to 2005.

Meropol et al^3^ state that the direct costs of cancer care include diagnostic tests, hospital and physician fees, and the cost of drug therapy. The high price of new drugs obscures other direct costs that are more difficult to enumerate. Although pathology and laboratory tests contributed to the highest number of services performed in our study, our analysis showed that the cost of medical supplies, such as the chemotherapy drugs themselves, contributes the most to cancer care spending. This observation is in line with analyses by Shih and colleagues^23,24^ that showed that targeted therapies accounted for 63% of all chemotherapy expenditures in 2011 and dominated anticancer drug spending. They estimated that although the general prescription drug Consumer Price Index grew at 3% per year during 2007 to 2012, mean targeted oral anticancer medication prices increased by nearly 12% per year, reaching $7719 per patient per month in 2012. Thus, cost seems to be magnified for locally advanced and systemic diseases that slowly progress over the course of years and require multiple costly systemic agents (eg, breast cancer).^25^

In many cases, increased spending results from hospitalization.^26,27,28^ In our analysis, level 3 to 4 outpatient hospital visits were the most commonly billed codes for all cancers. In a SEER-Medicare analysis from 2002,^27^ costs increased yearly for all cancers. In 2002, Medicare paid an annual mean of $39 891 for initial care costs for each lung cancer case and $41 134 for each colorectal cancer case, increases of $7139 and $5345 over the respective 1991 payments. Jacobson et al^29^ reported that outpatient visits were the main contribution to cost (approximately 60%-70% of the total costs) for patients with oral cancer. In 2014, the Agency for Healthcare Research and Quality estimated that 58% of all cancer expenditures were for outpatient visits, while hospital stays accounted for only 27% of the total.^30^ These reports corroborate our findings.

The present analysis includes both generic and brand-name drugs, without distinction. Previous analyses comparing effectiveness of the agents have been performed. Empirical studies between generic and brand-name drugs have not identified safety concerns in the US, Canada, the EU, and Japan, where regulations and enforcement are strong.^31,32^ Thus, we would anticipate generic drugs to have similar efficacy and likely lower cost than their brand-name counterparts.

The increasing share of cancer costs paid by private and Medicaid coverage highlight several factors in the changing insurance landscape. First, the Patient Protection and Affordable Care Act significantly increased Medicaid coverage of low-income patients with cancer, and removed some of the barriers to private coverage such as risk rating and preexisting condition exclusions.^33,34^ Second, as private payers tend to pay significantly higher rates for care, and as new high-cost cancer therapies reach the market, private payers inevitably pay much more both overall and relative to other payers.^35^ Third, cancer rates for colorectal cancer and human papillomavirus–related cancers are increasing in some populations of patients younger than 65 years, and thus private payers and Medicaid will pay relatively more for cancer care.^12^ Fourth, competing causes of death (eg, heart disease^36^ and stroke^37^) are replacing death from cancer among many sites; thus, there are many more long-term survivors,^19,38^ and therefore a larger time window for costs to accumulate. As commercial payers cover an increasing portion of cancer costs, our study helps to highlight the areas that may be most likely to be associated with this increase in spending.

### Limitations

This study has several limitations. First, it is unclear which procedures are the “most wasteful” from the current analysis. Claims data lack clinical detail, so one cannot tell the extent to which care is wasteful, and hence, costs might be reduced if wasteful care was eliminated. It is possible that outpatient spending is high, but it is always critical for patient care; meanwhile, unnecessary testing may be a better target for curtailing spending. More research is needed to find which services provide the lowest value to patients, which may not be the services that represent the highest spending. Identifying areas of high out-of-pocket spending may be of highest policy concern for limiting patient exposure to significant financial burden. This area, along with survival and quality of life, will be explored in future work.

MarketScan focuses largely on individuals employed in large firms, so it may not be fully representative of privately insured individuals and is not representative of patients with other types of insurance or those who are uninsured. In addition, the time since diagnosis and the modality of treatment being used are associated with the spending of cancer care. Previous work shows that advanced-stage diagnoses were generally more costly than early-stage diagnoses.^39^ However, cancer stage is not available in the MarketScan database.

## Conclusions

Among privately insured patients with cancer included in the MarketScan database in 2018, patients with breast, colorectal, and prostate cancers had the greatest number of services performed, with the plurality of these services attributable to pathology and laboratory tests. Medical supplies and nonphysician services contributed the most to the plurality of spending. Further research is needed to explore the extent to which these costs reflect unnecessary or low-value care.

## Notes

Supplement. **eTable 1.** Codes Associated With Each Category **eTable 2.** Most Common CPT/HCPCS Codes Organized by Procedure Group. Top 10 codes from each group are provided **eTable 3.** Costliest CPT/HCPCS Codes Organized by Procedure Group. Top 10 codes from each group are provided
