# Supplementary material for: Medical Service Use and Charges for Cancer Care in 2018 for Privately Insured Patients Younger Than 65 Years in the US
Source: JAMA Netw Open. 2021 Oct 6;4(10):e2127784. doi: 10.1001/jamanetworkopen.2021.27784 (PMC8495533; doi:10.1001/jamanetworkopen.2021.27784)
Supplement: Supplement. — eTable 1. Codes Associated With Each Category eTable 2. Most Common CPT/HCPCS Codes Organized by Procedure Group. Top 10 codes from each group are provided eTable 3. Costliest CPT/HCPCS Codes Organized by Procedure Group. Top 10 codes from each group are provided [file jamanetwopen-e2127784-s001.pdf]

Supplemental Online Content

Zaorsky NG, Khunsriraksakul C, Acri SL et al. Medical service use and charges for cancer care in 2018 for privately insured patients younger than 65 years in the US. *JAMA Netw Open*. 2021;4(10):e2127784. doi:10.1001/jamanetworkopen.2021.27784

eTable 1. Codes associated with each category

eTable 2. Most common CPT/HCPCS codes organized by procedure group. Top 10 codes from each group are provided

eTable 3. Costliest CPT/HCPCS codes organized by procedure group. Top 10 codes from each group are provided

This supplemental material has been provided by the authors to give readers additional information about their work.

© 2021 Zaorsky NG, et al. *JAMA Network Open*.

eTable 1: Codes associated with each category

Code	Alias	Description	Category
00100-01999	CPT_I_group1	Anesthesia	Anesthesia
10004-69990	CPT_I_group2	Surgery	Surgery
70010-79999	CPT_I_group3	Radiology Procedures	Radiology
0001U-0247U, 80000-89398	CPT_I_group4	Pathology and Laboratory Procedures	Pathology/Laboratory
90281-99607 (excluding 99091-99499)	CPT_I_group5	Medicine Services and Procedures	Medical services
99091-99499	CPT_I_group6	Evaluation and Management	Evaluation/Management
NA	CPT_II_group1	Composite Measures	Ignored
NA	CPT_II_group2	Patient Management	Ignored
NA	CPT_II_group3	Patient History	Ignored
NA	CPT_II_group4	Physical Examination	Ignored
NA	CPT_II_group5	Diagnostic/Screening Processes or Results	Ignored
NA	CPT_II_group6	Therapeutic, Preventive or Other Interventions	Ignored
NA	CPT_II_group7	Follow-up or Other Outcomes	Ignored
NA	CPT_II_group8	Patient Safety	Ignored
NA	CPT_II_group9	Structural Measures	Ignored
NA	CPT_II_group10	Non-Measure Category II Codes	Ignored
NA	CPT_III	New, emergent medical techniques.	Ignored
A*	HCPCS_II_groupA	Ambulance and Other Transport Services and Supplies, Medical And Surgical Supplies, Administrative, Miscellaneous and Investigational	HCPCS II
B*	HCPCS_II_groupB	Enteral and Parenteral Therapy	HCPCS II
C*	HCPCS_II_groupC	Other Therapeutic Procedures, Outpatient PPS	HCPCS II
NA	HCPCS_II_groupD	Dental services	Ignored
E*	HCPCS_II_groupE	Durable Medical Equipment	HCPCS II
G*	HCPCS_II_groupG	Procedures / Professional Services	HCPCS II

© 2021 Zaorsky NG, et al. *JAMA Network Open*.

H*	HCPCS_II_groupH	Alcohol and Drug Abuse Treatment	HCPCS II
J*	HCPCS_II_groupJ	Drugs Administered Other than Oral Method, Chemotherapy Drugs	HCPCS II
K*	HCPCS_II_groupK	Durable medical equipment (DME) Medicare administrative contractors (MACs), Components, Accessories and Supplies	HCPCS II
L*	HCPCS_II_groupL	Orthotic Procedures and services, Prosthetic Procedures	HCPCS II
M*	HCPCS_II_groupM	Miscellaneous Medical Services, Screening Procedures, Other Services, Episode of Care	HCPCS II
P*	HCPCS_II_groupP	Pathology and Laboratory Services	HCPCS II
NA	HCPCS_II_groupQ	Temporary Codes	Ignored
R*	HCPCS_II_groupR	Diagnostic Radiology Services	HCPCS II
NA	HCPCS_II_groupS	Temporary National Codes (Non-Medicare)	Ignored
NA	HCPCS_II_groupT	National Codes Established for State Medicaid Agencies	Ignored
NA	HCPCS_II_groupU	Coronavirus Diagnostic Panel	Ignored
NA	HCPCS_II_groupV	Vision Services, Hearing Services	Ignored

© 2021 Zaorsky NG, et al. *JAMA Network Open*.

eTable 2: Most common CPT/HCPCS codes organized by procedure group. Top 10 codes from each group are provided

Procedure Code	Procedure group	CPT Code Expanded Description	Total spending ($)	Total number of procedures	Average cost per procedure ($)	Total spending per cancer [Median (Q1-Q3)]	Total number of procedures per cancer [Median (Q1-Q3)]
00731	Anesthesia	Anesthesia procedures for upper GI endoscopy (00731)	14727152	30720	479	809008 (576633 - 1386196)	1734 (1282 - 2698)
00811	Anesthesia	Anesthesia procedures for lower intestinal endoscopy (00811)	10690469	25394	421	348794 (210321 - 544461)	815 (502 - 1274)
00812	Anesthesia	Anesthesia procedures for screening colonoscopy (00812)	8759807	22482	390	268524 (152282 - 566865)	690 (386 - 1444)
00400	Anesthesia	Anesthesia procedures on the integumentary (00400)	12512000	22251	562	159983 (85570 - 582038)	304 (160 - 1204)
00402	Anesthesia	Anesthesia procedures for breast reconstruction (00402)	24130182	20225	1193	95855 (19330 - 160248)	76 (22 - 152)
00840	Anesthesia	Anesthesia procedures for laparoscopy in lower abdomen (00840)	17130311	15963	1073	357356 (273637 - 674942)	380 (249 - 671)
01610	Anesthesia	Anesthesia procedures for shoulder and axilla (01610)	15330181	15217	1007	50438 (19891 - 140513)	58 (26 - 136)
00532	Anesthesia	Anesthesia for access to central venous circulation (00532)	8016258	14847	540	240621 (155308 - 559339)	413 (284 - 1087)

© 2021 Zaorsky NG, et al. *JAMA Network Open*.

00790	Anesthesia	Anesthesia procedures for laparoscopy in upper abdomen (00790)	17162883	14549	1180	592678 (444697 - 917369)	527 (426 - 801)
00320	Anesthesia	Anesthesia procedures for neck (00320)	13479372	13330	1011	188366 (67444 - 524152)	199 (86 - 568)
36415	Surgery	Collection of venous blood by venipuncture (36415)	23491603	1164882	20	825654 (592281 - 1723862)	52054 (30874 - 108652)
36591	Surgery	Collection of blood from implantable venous access device (36591)	11813909	94521	125	444817 (271731 - 1035167)	2933 (1972 - 8360)
11100	Surgery	Biopsy of skin, subcutaneous tissue, mucous membrane (11100)	7089961	55623	127	162864 (85748 - 492206)	1263 (656 - 3920)
43239	Surgery	Esophagogastroduodenoscopy with biopsy (43239)	29889349	48430	617	1432690 (1038163 - 2365573)	2322 (1742 - 3768)
45380	Surgery	Colonoscopy with biopsy (45380)	35791034	48400	739	1113023 (685098 - 2402619)	1527 (954 - 3124)
36561	Surgery	Insertion of centrally inserted central venous catheter (36561)	81592955	45213	1805	2575663 (1433675 - 6318127)	1489 (806 - 3540)
20610	Surgery	Arthrocentesis, aspiration and / or injection (20610)	4463208	36737	121	188193 (79605 - 312474)	1602 (679 - 2466)
17000	Surgery	Destruction (e.g. laser surgery) of premalignant lesions (17000)	2556036	36315	70	76658 (41227 - 158246)	1105 (519 - 2136)

© 2021 Zaorsky NG, et al. *JAMA Network Open*.

45385	Surgery	Colonoscopy with removal of tumor, polyp, or lesion (45385)	27420525	33812	811	949425 (507909 - 1669731)	1158 (662 - 2042)
45378	Surgery	Colonoscopy (45378)	23210421	31410	739	674388 (436877 - 1411014)	934 (638 - 1868)
74177	Radiology	Abdominal or pelvic CT with contrast material (74177)	170497289	243810	699	9039299 (4683927 - 12830692)	13413 (6670 - 18451)
77412	Radiology	Radiation treatment delivery, > 1 MeV; complex (77412)	144423099	230418	627	1662256 (1247334 - 3682163)	2730 (1986 - 5302)
71260	Radiology	Thoracic CT with contrast material (71260)	103139816	225244	458	4813137 (3132124 - 7679467)	10288 (6736 - 16950)
71046	Radiology	Radiological examination, chest; 2 views (71046)	15479934	207024	75	850737 (404452 - 1286775)	10883 (5804 - 17365)
77014	Radiology	CT guidance for placement of radiation therapy fields (77014)	23367868	198536	118	735808 (338995 - 2308392)	6240 (3133 - 19844)
71045	Radiology	Radiological examination, chest; single view (71045)	6460039	195033	33	288683 (192456 - 498669)	7515 (6698 - 15289)
77067	Radiology	Screening mammography, bilateral (77067)	20980476	141222	149	598762 (180182 - 1602002)	4083 (1202 - 10720)
77387	Radiology	Guidance for localization of target volume (77387)	32493553	133479	243	682910 (499992 - 1580680)	3168 (1918 - 11413)
77427	Radiology	Radiation treatment management, 5 treatments (77427)	41744012	126500	330	906565 (581562 - 3262389)	2704 (1636 - 9925)

© 2021 Zaorsky NG, et al. *JAMA Network Open*.

77386	Radiology	Intensity modulated radiation treatment delivery; complex (77386)	214024457	123355	1735	7704518 (5316931 - 13111702)	4281 (2868 - 7812)
85025	Pathology/Laboratory	Complete blood count and differential WBC count (85025)	48589171	1283261	38	1618730 (1120093 - 3948831)	46126 (41297 - 97000)
80053	Pathology/Laboratory	Comprehensive metabolic panel (80053)	60679140	1163093	52	2434686 (1922821 - 4520687)	45872 (35548 - 89217)
88305	Pathology/Laboratory	Surgical pathology, level 4 (88305)	76830361	438252	175	2578793 (1473750 - 5612480)	17070 (9892 - 35352)
80061	Pathology/Laboratory	Lipid panel (80061)	6685491	354841	19	250046 (158339 - 510756)	14071 (7544 - 25928)
83735	Pathology/Laboratory	Magnesium (83735)	6035166	285490	21	268161 (208226 - 526580)	12705 (10054 - 24457)
80048	Pathology/Laboratory	Basic metabolic panel (80048)	9972518	264189	38	425681 (318109 - 783563)	11259 (9736 - 23274)
84443	Pathology/Laboratory	Thyroid stimulating hormone (84443)	9369012	263694	36	449562 (170898 - 674326)	10851 (4276 - 16540)
83036	Pathology/Laboratory	HbA1C (83036)	3576495	217991	16	175368 (93474 - 261777)	10521 (4976 - 15678)
85027	Pathology/Laboratory	Complete blood count (85027)	5402953	210707	26	247090 (198209 - 386324)	10073 (6700 - 17653)
83615	Pathology/Laboratory	Lactate dehydrogenase (83615)	3501724	188625	19	108827 (61100 - 268950)	6009 (3532 - 14718)
97110	Medical services	Therapeutic exercises (97110)	36073414	556613	65	1460744 (653821 - 2637230)	20448 (10183 - 41939)
97140	Medical services	Manual therapy techniques (97140)	24406214	422178	58	839462 (294691 - 1305282)	14594 (6736 - 27905)

© 2021 Zaorsky NG, et al. *JAMA Network Open*.

96413	Medical services	Chemotherapy administration (96413)	166031915	404714	410	5834858 (4052312 - 11231055)	13899 (9883 - 26903)
96375	Medical services	Subcutaneous or intramuscular injection; additional push (96375)	61728242	281801	219	1887171 (1132107 - 5114306)	10611 (6482 - 21894)
96372	Medical services	Subcutaneous or intramuscular injection (96372)	19045159	215949	88	936807 (402197 - 1446127)	7786 (5446 - 19566)
96367	Medical services	Intravenous infusion; additional infusion (96367)	27737153	178554	155	1166635 (547256 - 2240025)	6901 (3520 - 13388)
93010	Medical services	12 leads ECG with interpretation and report (93010)	2992009	170295	18	151908 (109067 - 286292)	8242 (6208 - 16293)
98941	Medical services	Chiropractic manipulative treatment; spinal (98941)	5855075	161080	36	215519 (103707 - 475207)	6006 (2902 - 12970)
97112	Medical services	Therapeutic exercises; neuromuscular reeducation (97112)	6615768	155962	42	272288 (131135 - 517768)	5789 (3084 - 12122)
97530	Medical services	Therapeutic activities (97530)	6909694	143979	48	317351 (133481 - 566307)	5700 (2796 - 11701)
99214	Evaluation/ Management	Outpatient visit, level 4 (99214)	209600177	1543567	136	9036672 (5327136 - 17713953)	67907 (38888 - 129676)
99213	Evaluation/ Management	Outpatient visit, level 3 (99213)	114985287	1295497	89	4657040 (2540244 - 9139525)	53610 (28950 - 102010)
99232	Evaluation/ Management	Inpatient hospital, level 2 (99232)	37255339	413323	90	1865065 (1182404 - 3719238)	21474 (13266 - 41366)

© 2021 Zaorsky NG, et al. *JAMA Network Open*.

99233	Evaluation/Management	Inpatient hospital, level 3 (99233)	47251157	328765	144	2083656 (1321564 - 4422150)	14819 (9559 - 31656)
99215	Evaluation/Management	Outpatient visit, level 5 (99215)	59235385	291285	203	2336233 (1863333 - 5150898)	11868 (9268 - 26289)
99212	Evaluation/Management	Outpatient visit, level 2 (99212)	14139200	203905	69	619005 (347299 - 1009319)	8295 (4970 - 14967)
99396	Evaluation/Management	Preventative medicine reevaluation and management; 40-64 years (99396)	27823292	178179	156	848452 (426145 - 1960559)	5535 (2774 - 12508)
99203	Evaluation/Management	New office visit, level 3 (99203)	21807475	166743	131	850368 (459959 - 1888886)	6640 (3516 - 14504)
99204	Evaluation/Management	New office visit, level 4 (99204)	30069010	145966	206	1412379 (732580 - 2332641)	6899 (3552 - 11361)
99285	Evaluation/Management	Emergency department visit (99285)	83084920	139896	594	4291937 (2920720 - 7382634)	7762 (4938 - 12167)
J1100	HCPCS II	Dexamethasone sodium phosphate injection (J1100)	4639117	291571	16	204819 (111482 - 320442)	11652 (7216 - 18000)
J2405	HCPCS II	Ondansetron hydrochloride injection (J2405)	4089879	170780	24	168932 (130073 - 275734)	7570 (5398 - 11307)
J7050	HCPCS II	Normal saline infusion; 250 cc (J7050)	4395528	132574	33	163562 (119068 - 284520)	4209 (3549 - 9190)
G0463	HCPCS II	Outpatient clinic visit (G0463)	14498450	128778	113	557362 (480100 - 1062095)	5125 (3868 - 9926)

© 2021 Zaorsky NG, et al. *JAMA Network Open*.

J1642	HCPCS II	Heparin sodium injection (J1642)	2581325	125783	21	87476 (54798 - 197260)	4012 (2688 - 10005)
J2469	HCPCS II	Palonosetron injection (J2469)	53432783	120772	442	2039616 (1039666 - 3810478)	4624 (2106 - 8550)
J1200	HCPCS II	Diphenhydramine HCl injection (J1200)	2330652	116372	20	54235 (40779 - 206124)	3294 (2554 - 8228)
J3010	HCPCS II	Fentanyl citrate injection (J3010)	2417221	113736	21	114784 (74661 - 182131)	5123 (4233 - 7768)
G6002	HCPCS II	Ultrasonic guidance for placement of radiation therapy fields (G6002)	8039288	110105	73	154066 (105519 - 697351)	2695 (1576 - 10337)
J7030	HCPCS II	Normal saline infusion; 1,000 cc (J7030)	6766929	108845	62	301277 (205412 - 542943)	5103 (3410 - 9910)

© 2021 Zaorsky NG, et al. *JAMA Network Open*.

eTable 3: Costliest CPT/HCPCS codes organized by procedure group. Top 10 codes from each group are provided

Procedure Code	Procedure group	CPT Code Expanded Description	Total spending ($)	Total number of procedures	Average cost per procedure ($)	Total spending per cancer [Median (Q1-Q3)]	Total number of procedures per cancer [Median (Q1-Q3)]
00402	Anesthesia	Anesthesia procedures for breast reconstruction (00402)	24130182	20225	1193	95855 (19330 - 160248)	76 (22 - 152)
00790	Anesthesia	Anesthesia procedures for laparoscopy in upper abdomen (00790)	17162883	14549	1180	592678 (444697 - 917369)	527 (426 - 801)
00840	Anesthesia	Anesthesia procedures for laparoscopy in lower abdomen (00840)	17130311	15963	1073	357356 (273637 - 674942)	380 (249 - 671)
01610	Anesthesia	Anesthesia procedures for shoulder and axilla (01610)	15330181	15217	1007	50438 (19891 - 140513)	58 (26 - 136)
00731	Anesthesia	Anesthesia procedures for upper GI endoscopy (00731)	14727152	30720	479	809008 (576633 - 1386196)	1734 (1282 - 2698)
00320	Anesthesia	Anesthesia procedures for neck (00320)	13479372	13330	1011	188366 (67444 - 524152)	199 (86 - 568)
00400	Anesthesia	Anesthesia procedures on the integumentary (00400)	12512000	22251	562	159983 (85570 - 582038)	304 (160 - 1204)
00811	Anesthesia	Anesthesia procedures for lower intestinal endoscopy (00811)	10690469	25394	421	348794 (210321 - 544461)	815 (502 - 1274)
00865	Anesthesia	Anesthesia procedures for radical prostatectomy (00865)	9701341	7106	1365	31865 (9037 - 52794)	22 (8 - 39)

© 2021 Zaorsky NG, et al. *JAMA Network Open*.

00812	Anesthesia	Anesthesia procedures for screening colonoscopy (00812)	8759807	22482	390	268524 (152282 - 566865)	690 (386 - 1444)
36561	Surgery	Insertion of centrally inserted central venous cathether (36561)	81592955	45213	1805	2575663 (1433675 - 6318127)	1489 (806 - 3540)
19301	Surgery	Partial mastectomy (19301)	43090453	24831	1735	96498 (36267 - 301749)	59 (22 - 140)
19357	Surgery	Breast reconstruction with tissue expander (19357)	40698734	12698	3205	146597 (40911 - 305838)	48 (19 - 93)
45380	Surgery	Colonoscopy with biopsy (45380)	35791034	48400	739	1113023 (685098 - 2402619)	1527 (954 - 3124)
19303	Surgery	Complete mastectomy (19303)	35257320	17499	2015	101721 (43012 - 287107)	46 (22 - 126)
19083	Surgery	Ultrasound-guided breast biopsy (19083)	32218236	29747	1083	148343 (51237 - 368342)	158 (52 - 334)
38525	Surgery	Biopsy of deep axillary lymph nodes (38525)	30322559	27609	1098	91041 (50662 - 247898)	62 (32 - 223)
55866	Surgery	Laparoscopy / Surgical prostatectomy (55866)	29994376	9833	3050	87139 (43144 - 159816)	28 (8 - 75)
43239	Surgery	Esophagogastroduodenoscopy with biopsy (43239)	29889349	48430	617	1432690 (1038163 - 2365573)	2322 (1742 - 3768)
19380	Surgery	Revision of reconstructed breast (19380)	28098508	11359	2474	86557 (25484 - 178067)	36 (9 - 64)
77386	Radiology	Intensity modulated radiation treatment delivery; complex (77386)	214024457	123355	1735	7704518 (5316931 - 13111702)	4281 (2868 - 7812)

© 2021 Zaorsky NG, et al. *JAMA Network Open*.

74177	Radiology	Abdominal or pelvic CT with contrast material (74177)	170497289	243810	699	9039299 (4683927 - 12830692)	13413 (6670 - 18451)
78815	Radiology	PET with CT from skull base to mid-thigh (78815)	157292031	89398	1759	4584130 (3570155 - 15042148)	2551 (2064 - 8556)
77412	Radiology	Radiation treatment delivery, > 1 MeV; complex (77412)	144423099	230418	627	1662256 (1247334 - 3682163)	2730 (1986 - 5302)
71260	Radiology	Thoracic CT with contrast material (71260)	103139816	225244	458	4813137 (3132124 - 7679467)	10288 (6736 - 16950)
77385	Radiology	Intensity modulated radiation treatment delivery; simple (77385)	101317427	67198	1508	656220 (237976 - 940517)	365 (146 - 580)
77334	Radiology	Treatment devices, design, and construction; complex (77334)	71865344	91975	781	1712539 (991639 - 2984862)	2074 (1410 - 5064)
70553	Radiology	Brain MRI without contrast material, followed by contrast material (70553)	56046136	61727	908	1824987 (767937 - 3288253)	2169 (898 - 3694)
77295	Radiology	3-dimensional radiotherapy plan (77295)	55074951	35861	1536	1253884 (679417 - 2208902)	789 (428 - 1594)
77373	Radiology	Stereotactic body radiation therapy (77373)	51313378	9445	5433	2104696 (717975 - 3810732)	363 (156 - 732)
88305	Pathology/Laboratory	Surgical pathology, level 4 (88305)	76830361	438252	175	2578793 (1473750 - 5612480)	17070 (9892 - 35352)

© 2021 Zaorsky NG, et al. *JAMA Network Open*.

80053	Pathology/Laboratory	Comprehensive metabolic panel (80053)	60679140	1163093	52	2434686 (1922821 - 4520687)	45872 (35548 - 89217)
85025	Pathology/Laboratory	Complete blood count and differential WBC count (85025)	48589171	1283261	38	1618730 (1120093 - 3948831)	46126 (41297 - 97000)
88307	Pathology/Laboratory	Surgical pathology, level 5 (88307)	37056335	108916	340	1172312 (882099 - 1614931)	4770 (3502 - 5570)
88341	Pathology/Laboratory	Immunohistochemistry / Immunocytochemistry; additional stain (88341)	26259736	125413	209	1058352 (678649 - 2118239)	4796 (3346 - 11622)
81519	Pathology/Laboratory	Oncotype DX breast cancer assay (81519)	20628657	5388	3829	66555 (22119 - 114062)	18 (6 - 29)
88342	Pathology/Laboratory	Immunohistochemistry / Immunocytochemistry; initial stain (88342)	19431382	166583	117	621443 (421427 - 1362308)	5751 (4238 - 13622)
88360	Pathology/Laboratory	Morphometric analysis / Tumor immunohistochemistry (88360)	14024108	53273	263	159868 (113694 - 438708)	1000 (612 - 1806)
88185	Pathology/Laboratory	Flow cytometry; additional marker (88185)	13159577	20840	631	347049 (120222 - 403683)	493 (188 - 658)
81211	Pathology/Laboratory	BRCA1, BRCA2 gene analysis (81211)	12545138	9176	1367	198109 (97753 - 322883)	197 (108 - 304)
96413	Medical services	Chemotherapy administration (96413)	166031915	404714	410	5834858 (4052312 - 11231055)	13899 (9883 - 26903)
90999	Medical services	Dialysis (90999)	65923012	62297	1058	2786891 (1435781 - 5123929)	2313 (1208 - 5298)

© 2021 Zaorsky NG, et al. *JAMA Network Open*.

96375	Medical services	Subcutaneous or intramuscular injection; additional push (96375)	61728242	281801	219	1887171 (1132107 - 5114306)	10611 (6482 - 21894)
93306	Medical services	Transthoracic echocardiography (93306)	48549592	97725	497	1753148 (881191 - 2811135)	3660 (2076 - 6660)
97110	Medical services	Therapeutic exercises (97110)	36073414	556613	65	1460744 (653821 - 2637230)	20448 (10183 - 41939)
96417	Medical services	Chemotherapy administration; additional infusion (96417)	29429771	133621	220	779718 (484601 - 2310318)	4117 (2250 - 10495)
96367	Medical services	Intravenous infusion; additional infusion (96367)	27737153	178554	155	1166635 (547256 - 2240025)	6901 (3520 - 13388)
97140	Medical services	Manual therapy techniques (97140)	24406214	422178	58	839462 (294691 - 1305282)	14594 (6736 - 27905)
96416	Medical services	Chemotherapy administration; prolonged infusion (96416)	19395714	50559	384	342570 (137787 - 1264032)	993 (386 - 3112)
96372	Medical services	Subcutaneous or intramuscular injection (96372)	19045159	215949	88	936807 (402197 - 1446127)	7786 (5446 - 19566)
99214	Evaluation/Management	Outpatient visit, level 4 (99214)	209600177	1543567	136	9036672 (5327136 - 17713953)	67907 (38888 - 129676)
99213	Evaluation/Management	Outpatient visit, level 3 (99213)	114985287	1295497	89	4657040 (2540244 - 9139525)	53610 (28950 - 102010)
99285	Evaluation/Management	Emergency department visit, level 5 (99285)	83084920	139896	594	4291937 (2920720 - 7382634)	7762 (4938 - 12167)

© 2021 Zaorsky NG, et al. *JAMA Network Open*.

99215	Evaluation/Management	Outpatient visit, level 5 (99215)	59235385	291285	203	2336233 (1863333 - 5150898)	11868 (9268 - 26289)
99284	Evaluation/Management	Emergency department visit, level 4 (99284)	50936685	88926	573	2307357 (1716752 - 4632781)	3971 (3028 - 7823)
99233	Evaluation/Management	Inpatient hospital, level 3 (99233)	47251157	328765	144	2083656 (1321564 - 4422150)	14819 (9559 - 31656)
99232	Evaluation/Management	Inpatient hospital, level 2 (99232)	37255339	413323	90	1865065 (1182404 - 3719238)	21474 (13266 - 41366)
99204	Evaluation/Management	New office visit, level 4 (99204)	30069010	145966	206	1412379 (732580 - 2332641)	6899 (3552 - 11361)
99396	Evaluation/Management	Preventative medicine reevaluation and management; 40-64 years (99396)	27823292	178179	156	848452 (426145 - 1960559)	5535 (2774 - 12508)
99205	Evaluation/Management	New office visit, level 5 (99205)	25632174	84498	303	990176 (817869 - 2026415)	3325 (2602 - 6536)
J2505	HCPCS II	Pegfilgrastim injection (J2505)	460197547	65528	7023	11837459 (6083415 - 32510001)	1601 (814 - 4618)
J9355	HCPCS II	Trastuzumab injection (J9355)	421036199	77438	5437	2804081 (1310648 - 6860508)	494 (250 - 1216)
J9299	HCPCS II	Nivolumab injection (J9299)	283530907	29986	9455	6542596 (2501461 - 12347169)	710 (292 - 1388)
J9271	HCPCS II	Pembrolizumab injection (J9271)	237223963	17974	13198	7403222 (5887203 - 11535333)	565 (457 - 892)
J9035	HCPCS II	Bevacizumab injection (J9035)	203065997	40130	5060	2680522 (1990861 - 11052946)	648 (414 - 1952)

© 2021 Zaorsky NG, et al. *JAMA Network Open*.

J9306	HCPCS II	Pertuzumab injection (J9306)	196696010	24895	7901	558467 (188077 - 1220943)	82 (34 - 158)
J9310	HCPCS II	Rituximab injection (J9310)	164884243	19383	8507	2246860 (829257 - 2647375)	260 (100 - 316)
J9228	HCPCS II	Ipilimumab injection (J9228)	107939459	3880	27819	1939559 (1077537 - 3724995)	77 (47 - 176)
J0897	HCPCS II	Denosumab injection (J0897)	93171445	33561	2776	1285359 (940447 - 3159914)	472 (308 - 1058)
J9305	HCPCS II	Pemetrexed injection (J9305)	85312824	12094	7054	761234 (522236 - 1503690)	109 (78 - 208)

© 2021 Zaorsky NG, et al. *JAMA Network Open*.
